# Advancing the management of double hit lymphoma

**DOI:** 10.18632/oncotarget.20794

**Published:** 2017-09-09

**Authors:** Daniel J. Landsburg

**Affiliations:** Daniel J. Landsburg: Lymphoma Program, Abramson Cancer Center, University of Pennsylvania, Philadelphia, PA, USA

**Keywords:** lymphoma, B cell, genes, MYC, *in situ* hybridization

Double hit lymphoma (DHL), commonly defined as an aggressive B cell non-Hodgkin lymphoma (NHL) harboring rearrangement of *MYC* as well as *BCL2* and/or *BCL6*, has recently been formally categorized as “High grade B-cell lymphoma [HGBL], with rearrangements of MYC and BCL2 and/or BCL6” as per the 2016 revision of the World Health Organization (WHO) classification of lymphoid neoplasms. Patients with DHL can frequently present with adverse prognostic findings such as advanced clinical stage, elevated lactate dehydrogenase level and increased Ki67 expression on immunohistochemical staining [1, 2]; however, baseline clinicopathologic characteristics may not be predictive of the presence of double hit lymphoma in patients diagnosed with aggressive B cell NHL [3].

Initially-reported case series of DHL revealed poor clinical outcomes for patients with this disease regardless of therapy received [4, 5]. A subsequent multicenter retrospective analysis of over 300 DHL patients demonstrated improved progression free survival for patients treated with intensive front-line immunochemotherapy regimens as compared to those treated with standard rituximab-CHOP, although 2 year overall survival for all patients was only 49% and did not differ between front-line regimen received [2]. One strategy utilized to reduce the risk of relapse for patients with DHL has been consolidation with autologous stem cell transplantation (ASCT) in first complete remission (CR1); however, a recent multicenter retrospective analysis of over 150 DHL patients achieving CR1 showed a 3 year relapse free survival rate of 88% in patients treated with intensive front-line therapy, with no difference based upon receipt of ASCT. For those patients who did relapse, the median overall survival from the time of relapse was 8.6 months [6].

Given the overall poor prognosis of DHL, multiple novel agents are currently under investigation specifically for patients with *MYC* rearranged or MYC protein overexpressing aggressive lymphomas, including those with DHL, as either monotherapy or in combination with immunochemotherapy. These include the dual PI3 kinase/histone deacetylase inhibitor CUDC-907 (NCT02674750), the aurora kinase inhibitor alisertib (NCT02700022) and the immunomodulatory agent lenalidomide (NCT02213913). While use of these agents has led to disease response in preclinical models, their efficacy in the MYC-“altered” lymphoma, and specifically DHL, patient population remains to be seen.

Based on variation in clinical presentation, as well as response to both standard and intensive front-line immunochemotherapy regimens experienced by patients diagnosed with DHL, it is apparent that biologic variation exists even within this subset of aggressive B cell NHL. This is supported by at least one study in which sequencing of a panel of genes frequently mutated in Burkitt lymphoma performed on tissue specimens from patients with DHL did not identify a common mutation pattern [7]. It is possible that application of a broader next generation sequencing panel to diagnostic tissue specimens of a previously-treated cohort of DHL patients may identify abnormal gene signatures associated with clinical outcomes. Alternatively, a more focused approach could include assessing DHL tissue specimens for alterations of genes or proteins involved in pathways regulated by *MYC*, such as the Arf-p53 tumor suppressor pathway or that of mitochondrial-induced apoptosis regulated by the Bcl-2 family of proteins [8], which may ultimately lead to a more focused use of small molecule inhibitors and other novel therapeutics in DHL patients with specific molecular profiles.

More widespread utilization of cytogenetic testing to detect rearrangements in *MYC*, *BCL2* and *BCL6* in the evaluation of aggressive B cell NHL tissue specimens has led to increased recognition of cases of DHL, and consequently, appreciation of the varied clinical outcomes experienced by these patients . For this disease defined by a set of genetic abnormalities, it now seems prudent to search for additional molecular alterations that may inform prognosis, and more importantly, allow for the rational use of targeted therapies in an effort to increase in clinical benefit and minimize unwarranted toxicity in patients with DHL.
